# Ocular knowledge and practice among type 2 diabetic patients in a tertiary care hospital in Bangladesh

**DOI:** 10.1186/s12886-017-0560-x

**Published:** 2017-09-19

**Authors:** Kazi Rumana Ahmed, Fatema Jebunessa, Sharmin Hossain, Hasina Akhter Chowdhury

**Affiliations:** 1 Department of Health Promotion and Health Education, Bangladesh University of Health Sciences, 125/1, Darus Salam, Mirpur-1, Dhaka, Bangladesh; 2 School of Health and Rehabilitation Sciences, The University of Queensland, Brisbane, Australia; 3 Department of Biochemistry and Cell Biology, Bangladesh University of Health Sciences, 125/1, Darus Salam, Mirpur-1, Dhaka, Bangladesh; 4 Department of Biostatistics, Bangladesh University of Health Sciences, 125/1, Darus Salam, Mirpur-1, Dhaka, Bangladesh

**Keywords:** Type 2 diabetes, Ocular knowledge, Practice, Hospital-based study, Bangladesh

## Abstract

**Background and aims:**

Diabetes mellitus is likely to have a major effect on vision, and adequate knowledge of its ocular manifestations is of substantial importance to diabetic patients. The study aimed to assess the ocular knowledge and practices among Type 2 diabetic patients of Bangladesh.

**Methods:**

This cross-sectional study included 122 diabetic patients from the outpatient department (OPD) of the apex diabetic healthcare hospital of the country under the sponsorship of the Diabetic Association of Bangladesh (BIRDEM). A questionnaire was used for collecting data on knowledge on and practices relating to diabetes mellitus with particular emphasis on ocular issues. A predefined score was used for categorizing levels of knowledge and practices as poor, average, and good.

**Results:**

Of the 122 respondents, 63%, 55%, 40%, 44%, and 30% reported, blindness, retinopathy, cataracts, glaucoma, and double vision respectively, as complications of diabetes mellitus. About 50% were aware of the need for eye screening for people with the complications. Only 8% monitored their blood glucose levels daily, 15% monitored weekly, and 10% reported checking their blood pressure daily and 43% took their medications as prescribed. The level of diabetic knowledge was poor, moderate and good, respectively, among 24%, 56%, and 20% of the respondents, whereas the practice standards showed that 47%, 31%, and 22% had poor, average, and good levels respectively. The knowledge score was significantly associated with the practice score (*r* = 0.460, *p* = 0.001).

**Conclusion:**

The results indicate that the ocular knowledge and practices among diabetic patients attending a tertiary-care hospital in Bangladesh is average. Health and eye-care practitioners need to expand diabetic health education and promotion among diabetic patients.

## Background

Diabetes mellitus (DM) is a growing global epidemic and leading cause of ocular complications, eye complications, and eye diseases, such as cataract, retinopathy, glaucoma, double vision, macular degeneration, and blindness [1]. The longer duration of DM is associated with ocular complications, resulting in visual impairment and blindness.

The rising trend of non-communicable diseases, especially DM, along with lifestyle-related factors, such as diet, sedentary lifestyle, and smoking [2, 3], are the likely causes of reversible vision loss in Bangladesh. Results of studies in Bangladesh among persons, aged ≥30 years, showed that 22.6% were suffering from low vision [4], and 22.1% were myopia [5].

Kknowledge on the prevention and risk factors of ocular complications is essential to prevent vision loss among DM patients. Although the majority of diabetes patients are aware that diabetes can cause eye diseases, their attitude and practice is not at the desired level, which need to be improved [6]. The need for greater awareness of prevention, diagnosis, control of risk factors and disease management is well-documented in various studies. [7, 8]. Results of a population-based study on eye diseases in a rural district in Bangladesh showed that 58% of participants did not have any idea about their vision loss, which could be prevented [3]. Results of a community-based study among diabetic patients showed that males were more likely than females to know that diabetes causes eye diseases (18% vs 13%), and 38% of patients with at least SSC level of education knew that diabetes causes eye diseases compared to those with no schooling (9%) *p* = 0.001 [9]. Although the respondents could define diabetes, they had poor knowledge on ocular complications and also had poor attitude and practice relating eye care.

To the best of our knowledge, no hospital-based study was conducted in Bangladesh to understand the knowledge and practices of diabetic patients relating to eye diseases. The presents study was therefore undertaken to assess the level of ocular-related knowledge and practices among type 2 diabetic patients in a tertiary-care hospital in Bangladesh.

## Methods

Using a cross-sectional study design, 122 adult diabetic patients were included from the outpatient department of the Bangladesh Institute of Research & Rehabilitation in Diabetes, Endocrine and Metabolic Disorders (BIRDEM), which is an apex diabetic healthcare hospital of the country under the sponsorship of the Diabetic Association of Bangladesh [10]. Detailed profiles of diabetic patients were obtained from the patient’s guidebook in the hospital. Furthermore, an ophthalmologist confirmed eye complications due to diabetes.

### Study population

All the registered diabetic patients who visited the ophthalmic OPD during were conserved for inclusion in the study. A pilot study was conducted in the ophthalmic OPD among 18 diabetic patients who did not take part in the final study. All the queries arising from the pilot study were addressed before the final study was conducted.

### Sample-size

In this study, the prevalence of knowledge on eye disorders among DM patients was considered 75.62% [6] for determining the sample-size using the following formula: n = z2q/r2p. Thus, the sample-size was approximately 125.

### Sampling technique

Systematic sampling method was followed to select subjects for the study. Every 10th DM patient from each ophthalmologist patient’s registry book was selected to participate in the study till the sample-size of the study had achieved.

### Questionnaire

Most (97.5%) of the participants answered the questionnaire voluntarily, and confidentiality was maintained throughout the study. A face-to-face interview was conducted by trained interviewers using a two-part semi-structured questionnaire. The first part of the questionnaire contained socio-demographic information (i.e. age, gender, socioeconomic status, education) of the patients, duration of DM, and complications due to diabetes. The second part contained 11 questions, of which seven questions were about knowledge of patients on ocular complications due to DM and four were related to ocular self-care practices of the patients. Knowledge was defined as the understanding of information regarding ocular knowledge, and practice was defined as the understanding of information regarding ocular practice relating to diabetes.

### KAP scoring

The maximum attainable scores for knowledge and practices were 7 and 4 in that order, and the minimum score was ‘0’. The levels of (poor, average, and good) knowledge and the practice scores were considered as (mean ± 1 SD) [11]. The levels of knowledge and practices were categorized based on each respondent’s attainable score.

### Statistical analysis

At the end of interviews, the researchers checked and verified data. Descriptive statistics was used for analyzing the sociodemographic characteristics and knowledge of the respondents on different aspects of ocular complications due to diabetes. Pearson’s correlation was performed to determine the association between the knowledge and the practices of the participants. The SPSS software (version 16.0) was used for analyzing all the data.

### Ethical aspects

The ethics review committee of the Bangladesh University of Health Sciences approved the study protocol.

## Results

The mean (±SD) age of the 122 participants was 57 ± 8 years. Of them, a male (55%) preponderance was observed. The majority (62%) of the respondents had higher secondary education and above. Around 30% were from a high-income group. Forty-six percent were engaged in different types of activities (Table 1).

**Table 1:** Characteristics of the study subjects (*n* = 122)

Variables	n (%)
Age (yrs)	
≤ 45	10 (8.2)
46–65	91 (74.6)
> 65	21 (17.2)
*Mean ± SD*	*57.57 ± 8.76*
Gender	
Male	67 (55)
Female	55 (45)
Religion	
Muslim	116 (97)
Hindu	4 (3)
Marital Status	
Married	119 (98)
Unmarried	3 (2)
level of Education	
Primary to Secondary	47 (38)
Higher Secondary-Graduate & above	75 (62)
Occupation	
Unemployed	66 (54)
Employed	56 (46)
Socio-economic status (BDT*)	
Low income (≤10,000)	47 (38.5)
Middle income (10001–20,000)	39 (32)
High income (>20,000)	36 (29.5)
Duration of diabetes (yrs)	
Mean ± SD	9.75 ± 3.15
Diabetes status	
Controlled Group	48 (39)
Uncontrolled Group	74(61)

Results are expressed number (percentage) and mean ± SD; *BDT = Bangladeshi Taka

The majority (63%) of the respondents pointed out blindness as an eye complication due to diabetes, followed by diabetic retinopathy (55%) and other eye complications. Forty-nine percent had concept about the necessity of regular eye screening. In reply to a question on the frequency of the test, 35% indicated to perform it in every 6–12 months (Table 2).

**Table 2:** Responses of different questions related to ophthalmic knowledge among patients with diabetes

Ophthalmic knowledge/Indicator	Correct Answer [n (%)]
Knowledge of diabetic eye complication*	
Blindness	65 (63)
Diabetic retinopathy	67 (55)
Cataracts	49 (40)
Glaucoma	54 (44)
Double vision	37 (30)
Knowledge of Eye Screening in diabetes mellitus	
Yes, to eye screening	59(49)
Frequency of Eye screening	
Every 6–12 months	43 (35)

Results are expressed as number (%)

*Multiple Responses

On eye-care practices, only 8% monitored their blood sugar daily; around 10% checked their blood pressure daily; and 43% always took medications as prescribed by the physicians. Around 37% visited an eye specialist once a year to prevent vision loss and other diabetes-associated eye complications (Table 3).

**Table 3:** Summary results of different types of self–care practices with diabetes

Practice related variables	Percentage (%)
Monitor blood glucose	
Daily	10 (8)
Weekly	18 (15)
Monthly	45 (37)
Never	49 (40)
Blood pressure monitor	
Daily	12 (10)
Weekly	4 (3)
Monthly	49 (40)
Never	57 (47)
Adherence to medication	
Regular	53 (43)
Irregular	69 (57)
Visiting an eye specialist	
Every 6–12 months	13 (11)
Every year	45 (37)
Felt problem with vision	64 (52)

Results are expressed as number (%)

**Fig. 1:**
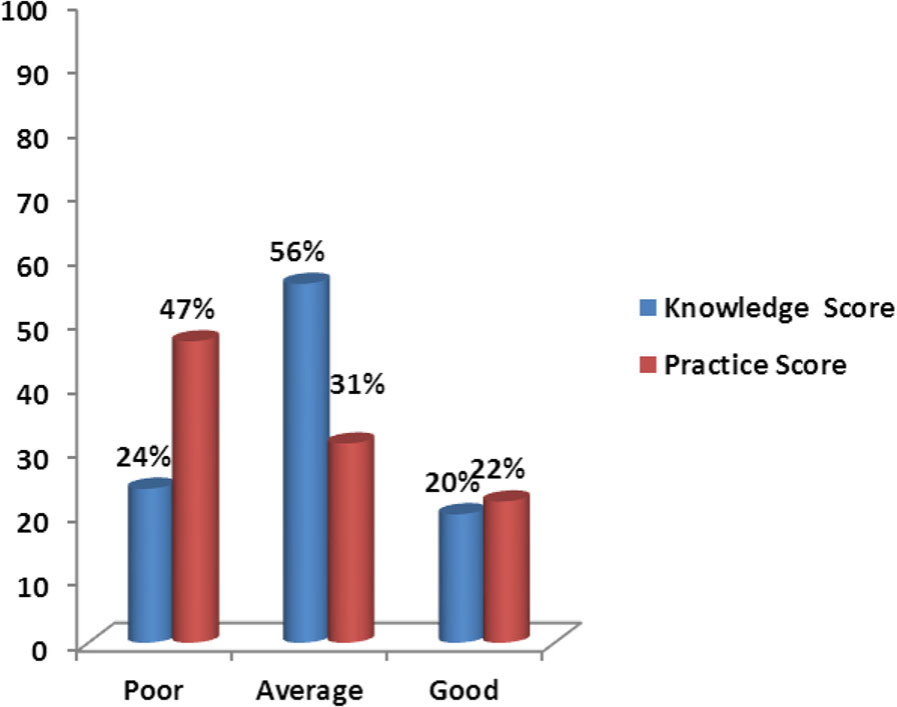
Total knowledge and practice scores among diabetes patients regarding different questions on ophthalmic knowledge and practices

**Fig. 2:**
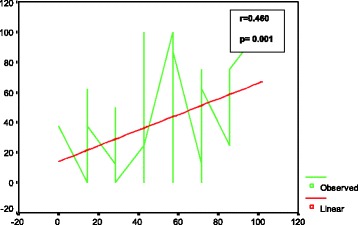
Association of total knowledge score with total practice score

The overall level of knowledge was poor, average, and good, respectively, among 24%, 56%, and 20% of the patients. Regarding practices, the corresponding values were poor in 47%, average in 31%, and good in 22% of the participants (Fig.1) respectively. On Pearson’s correlation, a significant positive association (*r* = 0.460, *p* = 0.001) was found between the knowledge and the practice score (Fig.2).

## Discussion

Diabetes mellitus is the most widespread public-health challenge that has been confronting the present century. Its timely management and routine eye examinations can decrease or delay its complications [6]. Our main objective was to investigate the levels of knowledge of the diabetic patients about ocular issues.

The findings of our study re-assert the gaps between the ophthalmic knowledge and the practices among the diabetic patients in Bangladesh. The period of onset of ophthalmic complications determines the type of diabetes an individual suffers [12]. Approximately 25% of type 2 diabetic patients develop some degree of diabetic retinopathy before their diagnosis. [13, 14].

This study has shown that 63%, 55%, and 40% of the patients had adequate knowledge, respectively, on the complications of blindness, retinopathy, and cataract associated with DM. Thirty percent had adequate knowledge on the complications of double vision associated with this condition. Results of a similar study in Oman revealed that 72.9% of diabetic patients had adequate knowledge on ophthalmic complications [15], which is much more than that in our study. Results of other studies showed that over 95% of subjects were concerned about DM and its ophthalmic consequences [16, 17]. Contrary to our findings was observed in a study conducted in Ghana [12]. Our study focused on insufficient practices (irregular monitoring, non-adherence to lifestyle modification, and medical advices) of the patients relating to regular monitoring of blood glucose and blood pressure, adherence to medications, and visiting an eye specialist. However, in our study, only 37% of the patients visited the health professionals (physician and ophthalmologist) for regular eye examination annually, and 52% visited an eye specialist only when they had a vision problem, suggesting that most diabetic patients are not aware that eye complications can be present without any symptom, particularly in the early stages of the condition [18]. This view is supported by the fact that another study reported that only 19.5% of diabetic patients had their last ocular examinations within a year, and 34.5% had never any eye examination following the diagnosis of their DM [12]. These results compare well with those reported in Tanzania [19] and South Africa [20]. This finding suggests that lack of knowledge is a major barrier to eye care in Bangladesh. It may be due to the patients said that it is important to have a regular eye examination as per the advice of their physicians. Whether or not this accurately reflects the content of physician-patient discussions in this tertiary-care setting, it clearly indicates that optimal communication of an important piece of diabetic health education does not occur.

Inadequate knowledge on eye care among our patients resulted in the low uptake of eye care compared to participants in the Durban study [20]. However, knowledge of ocular complications and management options were low in our study subjects, which is similar to results obtained in Baltimore, USA [21]. A positive correlation between the knowledge and the practices was noted in this study. The information gap about eye diseases may be attributed to poor knowledge on ocular complications and poor eye-care practices relating to examination of eye complications due to DM. The results of our study also showed that the majority of patients were aware of the need for eye screening but eye-care practices were not at the desired level.

Based on the findings of the present study, it is recommended that the diabetic patients go for at least an annual comprehensive eye examination to facilitate early diagnosis and management of visual disorders associated with this condition to avoid visual impairment and blindness.

## Strengths and limitations

All the diabetic patients in the present study were attending the tertiary-care hospital of BIRDEM for diabetic care. BIRDEM sponsored by the Diabetic Association of Bangladesh and being an apex diabetic healthcare hospital of the country, it receives DM patients from almost all over Bangladesh, including affiliated associations of the Diabetic Association of Bangladesh (BADAS). Therefore, data generated from this hospital generally reflect the general diabetic population of the country. However, since the sample was drawn from only one hospital to explore the eye diseases of DM patients, it is a limitation of the study.

## Conclusions

The results of the present study have shown that diabetic patients in this facility had average knowledge but had poor eye-care practice. The results suggest the need for health education for their eye-care practices to reduce ocular complications in diabetic patients.

## Abbreviations

BIRDEM: Bangladesh Institute of Research and Rehabilitation in Diabetes, Endocrine and Metabolic Disorders
DM: Diabetes Mellitus
KAP: Knowledge, Attitudes and Practice
OPD: Out Patient Department
SD: Standard Deviation
SPSS: Statistical Package for Social Science
